# Wild mice with different social network sizes vary in brain gene expression

**DOI:** 10.1186/s12864-020-06911-5

**Published:** 2020-07-22

**Authors:** Patricia C. Lopes, Barbara König

**Affiliations:** 1 Schmid College of Science and Technology, Chapman University, Orange, CA USA; 2 Department of Evolutionary Biology and Environmental Studies, University of Zurich, Zürich, Switzerland

**Keywords:** Neurogenomics, Transcriptomics, Dopamine, X-chromosome inactivation, Extracellular matrix, Sex differences, Social interactions, Hippocampus, Hypothalamus, Prefrontal cortex

## Abstract

**Background:**

Appropriate social interactions influence animal fitness by impacting several processes, such as mating, territory defense, and offspring care. Many studies shedding light on the neurobiological underpinnings of social behavior have focused on nonapeptides (vasopressin, oxytocin, and homologues) and on sexual or parent-offspring interactions. Furthermore, animals have been studied under artificial laboratory conditions, where the consequences of behavioral responses may not be as critical as when expressed under natural environments, therefore obscuring certain physiological responses. We used automated recording of social interactions of wild house mice outside of the breeding season to detect individuals at both tails of a distribution of egocentric network sizes (characterized by number of different partners encountered per day). We then used RNA-seq to perform an unbiased assessment of neural differences in gene expression in the prefrontal cortex, the hippocampus and the hypothalamus between these mice with naturally occurring extreme differences in social network size.

**Results:**

We found that the neurogenomic pathways associated with having extreme social network sizes differed between the sexes. In females, hundreds of genes were differentially expressed between animals with small and large social network sizes, whereas in males very few were. In males, X-chromosome inactivation pathways in the prefrontal cortex were the ones that better differentiated animals with small from those with large social network sizes animals. In females, animals with small network size showed up-regulation of dopaminergic production and transport pathways in the hypothalamus. Additionally, in females, extracellular matrix deposition on hippocampal neurons was higher in individuals with small relative to large social network size.

**Conclusions:**

Studying neural substrates of natural variation in social behavior in traditional model organisms in their habitat can open new targets of research for understanding variation in social behavior in other taxa.

## Background

Maintenance of social ties involves trade-offs. While group living may facilitate finding sexual partners and promote cooperation in acquiring food, in offspring care and in protection against predators, it imposes conflicts in the form of competition for sexual partners and for resources [1]. Nonetheless, in several species of group-living mammals, maintenance of affiliative social ties is positively correlated with fitness outcomes in ways that are not yet fully understood [2]. Also, in humans, social interactions impact health outcomes [3–7]. Even if social interactions may be positive, intra-specific variation in social interaction traits is widespread in vertebrates [8, 9]. Taken to an extreme, impaired social behavior in humans is considered a disorder, and characterizes disabilities with very high incidence such as autism spectrum disorder and schizophrenia [10]. Understanding what neural mechanisms are associated with intra-specific variation in social behavior is therefore critically important from both a fundamental and applied perspective.

The last twenty years have seen a lot of progress in the understanding of the neural circuits, neuropeptides and neuromodulators involved in vertebrate social behavior [11–15]. Even in the light of all of this progress, it is important to note, however, that the social environment is one of the most unpredictable environments animals face, given that it is composed of several interactive agents [16, 17]. Paradoxically, we usually study the neurobiology of mammalian social behavior in somewhat simplified settings, using inbred animals, housed in conditions that are likely to prevent them from displaying their natural repertoire of behavioral and physiological responses [18]. In laboratory studies, animals are presented with an environment where the consequences of behavioral and physiological responses for survival may not be as severe as in a natural environment; moreover, the level of sterility and standardization may obscure certain responses (e.g., [19, 20]) or not apply to even slight deviations of the environmental conditions tested [21, 22]. This has important implications for the translational value that animal models have for neuropsychiatric disorders [23]. Recently, there have been a number of calls for studies that can integrate the proximate mechanisms underlying social behavior with their adaptive function [16, 17, 24]. In part, this integration can come from studying traditional model organisms in their natural environment. The challenge here is that many animals are difficult to observe in the wild, making detailed behavioral quantifications impractical.

There are many reasons that could lead to differences in social interaction patterns in adult animals, including developmental or early-life experiences (e.g., [25–27]), genetically determined social behavior differences (e.g., [28]), or current experiences (e.g., social defeat, [29]) (see [30] for an in-depth discussion of possible mechanisms leading to social plasticity). Regardless of the underlying cause of variation in frequency of social interactions, studies using complex group settings still find biological correlates of social behavior. In one study in fruit flies (*Drosophila melanogaster*), behavioral differences between individuals obtained through automated tracking of groups of flies were found to be consistent and able to accurately predict sex and genotype [31]. A study in wild house finches (*Haemorhous mexicanus*) found that exploratory and social behaviors were linked to stress physiology [32]. When large groups of male laboratory mice (*Mus musculus*) where studied in large structured lab enclosures, the number of ties those mice directed at other mice was negatively associated with hippocampal gene expression levels of a neural plasticity gene (DNMT1) [33]. A study in captive prairie voles (*Microtus ochrogaster*) maintained in semi-natural enclosures indicated that variation in vasopressin receptor 1A (V1aR) in particular brain regions may be linked to differences in sexual fidelity in males [34]. While much of the focus of nonapeptide (oxytocin, vasopressin, their homologues, and receptors) research has been on male-female sexual bonds and parent-offspring bonds [35, 36], adult individuals of many species form bonds that are unrelated to sexual or parental interactions, for instance, during the non-reproductive season, and these bonds impact fitness outcomes.

These studies indicate that, even with the noise that underlies studying complex social behaviors of animals in complex social and environmental settings, patterns of social interactions can be linked to genotypic and/or physiological differences. Recently, König and others have optimized an automated system that remotely collects continuous information on the social interactions of > 90% of a population of wild house mice in Switzerland [37]. We leveraged this novel setup to detect mice that consistently had social network sizes at opposite ends of the social network size distribution in a free-ranging population living in a barn with unlimited access to food. We then used RNA-seq to determine what neural differences in gene expression could be associated with these extreme differences. Different from experimental setups where researchers exposed animals to different aggregation treatments (group versus single housing [38];) in our study animals were free to determine their preferred association patterns, including being able to leave the population altogether. By following animals in a complex, natural setting, this study pushes the boundaries of how the neurogenetic underpinnings of social behavior are studied, with far-reaching implications for the understanding of human disorders that involve impairments of social interactions.

## Results

To obtain animals with contrasting social network sizes, we sampled individuals at both tails of a distribution of egocentric social network size for the population (number of different partners encountered in nest boxes per day) during the non-breeding season. In our population, social network size cannot be explained solely by time spent in nest boxes (*F*_1,393_ = 1.224, *p* = 0.2692, *r*^*2*^ = 0.0031; Figure S1A) or activity related to going in and out of nest boxes (*F*_1,393_ = 0.3459, *p* = 0.5568; *r*^*2*^ = 0.00088; Figure S1B). Repeatability (R) of social network size for mice in our population during the non-breeding season is high, R [95% confidence interval] = 0.9 [0.874, 0.914], *p* < 0.01. The mean social network size for animals in the population was (mean ± STD) 26 ± 8.6 partners/day. Sampled animals with large social network size had mean social network size values of 38.1 ± 2.5 and 32.5 ± 1.3 for females and males, respectively, while animals with small social network size had mean values of 9.5 ± 1.3 and 8.7 ± 2 for females and males, respectively. Males with large social network sizes therefore approached +1STD of the population mean (34.6 partners/day) but were not above it. Body mass of samplesd animals (Figure S2) was not different due to the social network size (F = 0.787, d.f. = 1, *p* = 0.38), nor due to a social network size by sex interaction (F = 0.6, d.f. = 1, *p* = 0.44), but differed between the sexes (F = 6.66, d.f. = 1, *p* = 0.016), with males (29.23 g ± 1.02) being heavier than females (25.68 g ± 0.91). RNA extracted from specific brain regions from these animals was then used for RNA-seq.

**Fig. 1:**
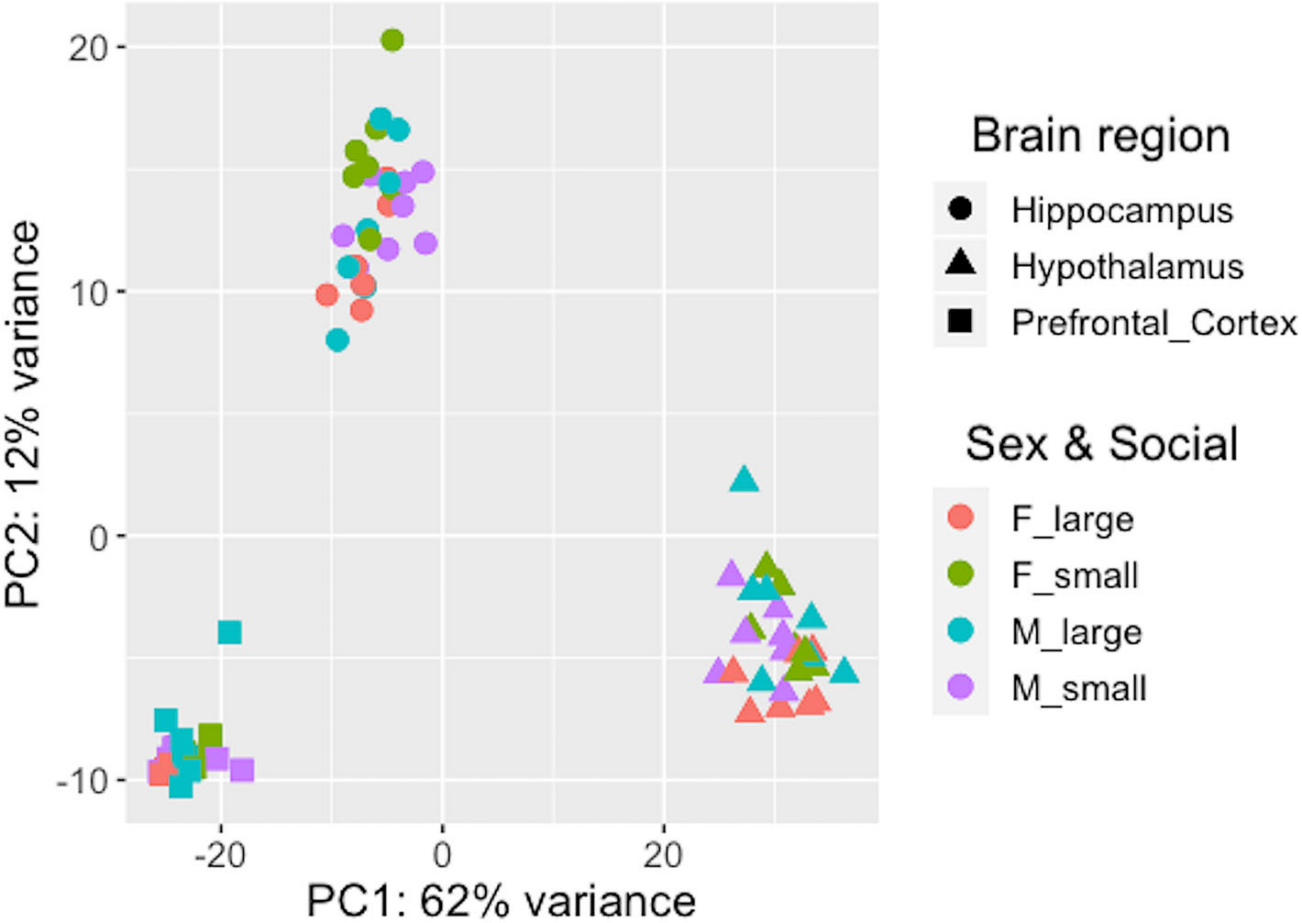
Principal Component Analysis of all mapped genes in three brain regions of free-ranging house mice of different sex and social network size. In the legend, F stands for female and M for male, and large or small for large or small social network size

An average of 94.5% clean reads were mapped to the reference genome for the mouse (Table S1). A principal component analysis of normalized read counts for all mapped genes shows that each of the brain regions (prefrontal cortex, hypothalamus and hippocampus) extracted from different animals cluster together (Fig. 1).

**Fig. 2:**
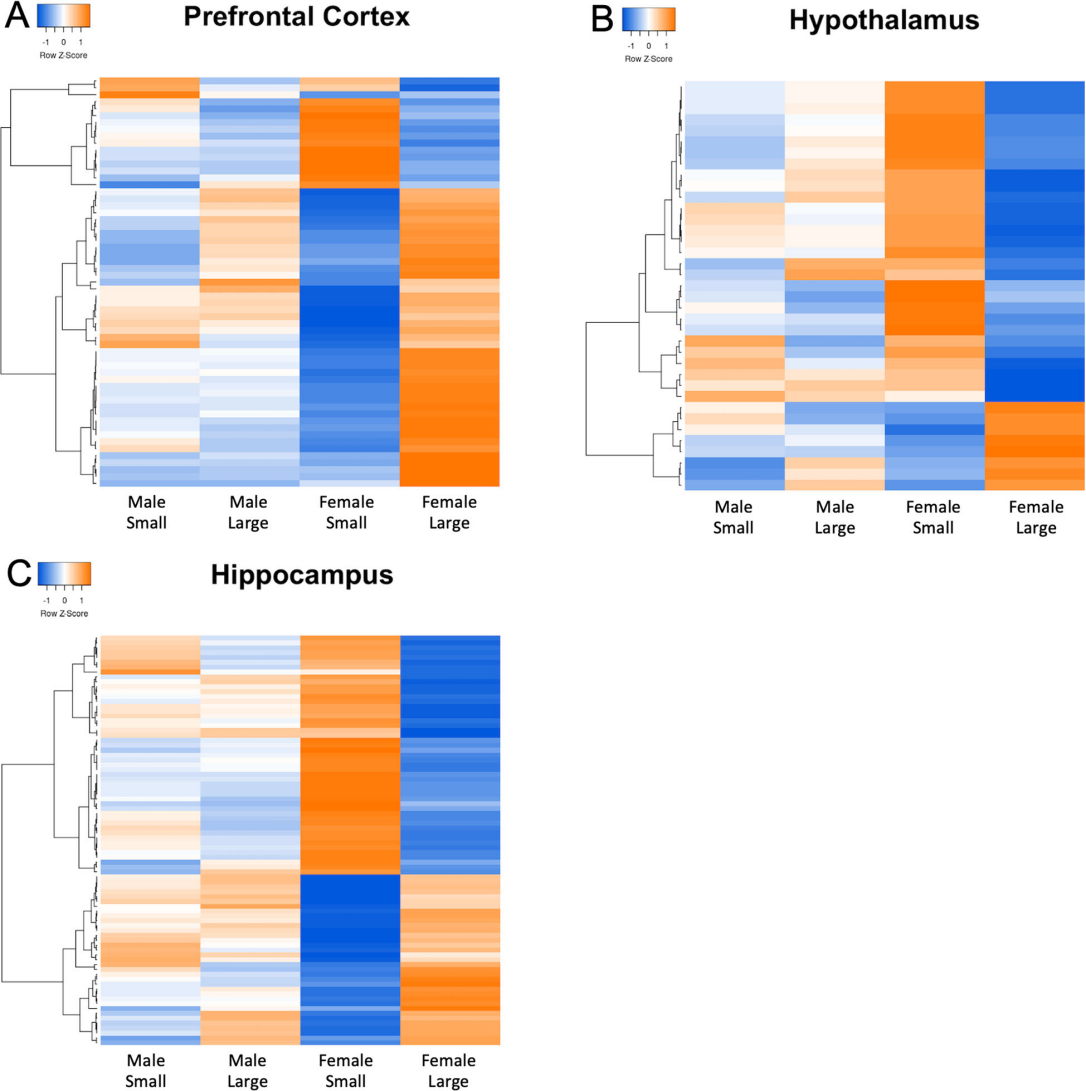
Heatmaps depicting all differentially expressed genes between females with large and small social network sizes in the prefrontal cortex (**a**), hypothalamus (**b**), and hippocampus (**c**). Male expression levels are also represented for comparison. The y-axis dendrogram represents the clustering of the rows (mean of normalized read counts for each differentially expressed gene) using Pearson distance. Blue color indicates lower and red color higher expression levels

Females had much larger numbers of differentially expressed genes between the social network size extremes than males (Table S2). Taking the subset of genes that were differentially expressed between females with small and large social network sizes in each of the brain regions and plotting male expression levels for those genes, it is possible to visualize the strong differences between females for each brain region (Fig. 2).

**Fig. 3:**
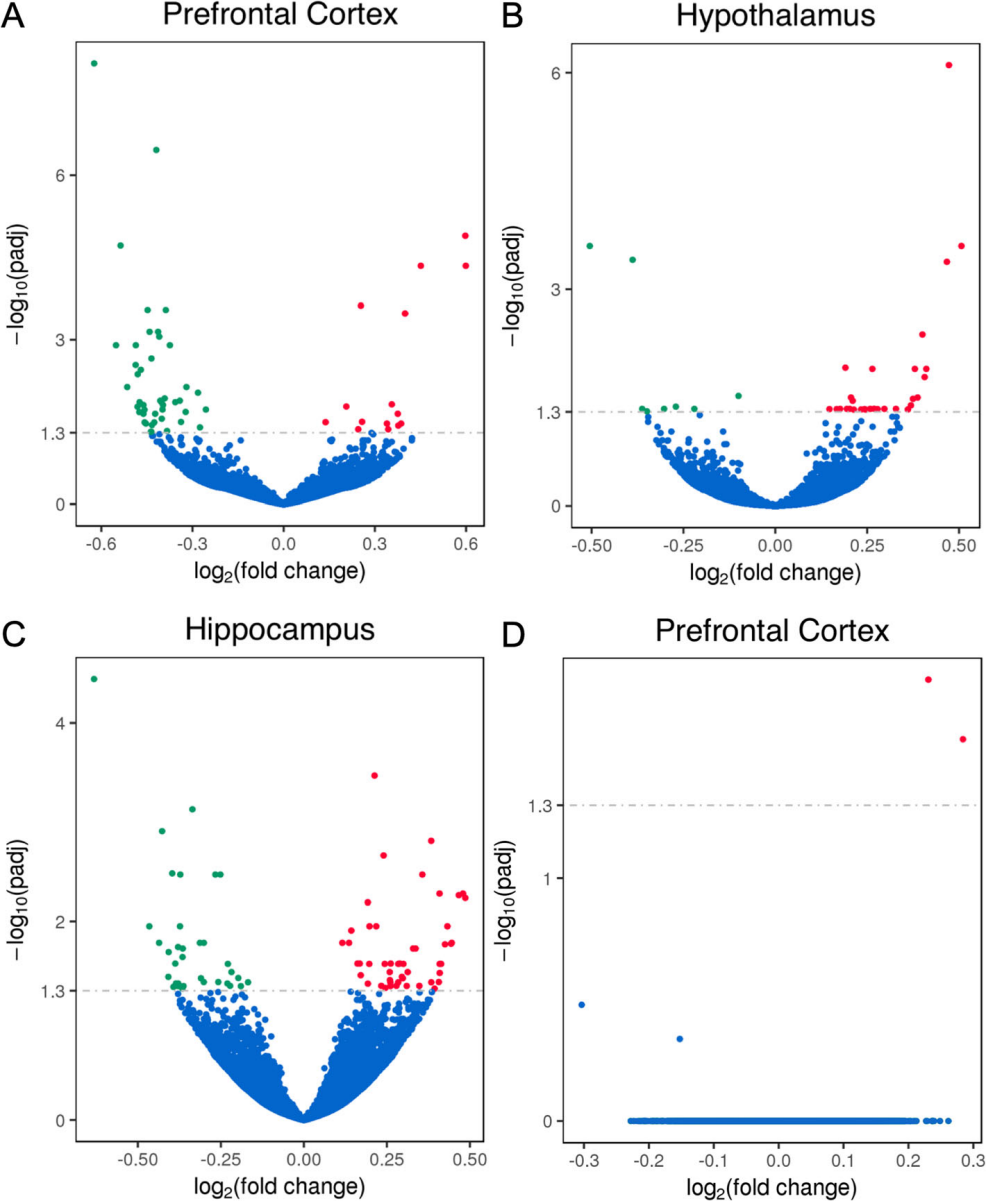
Volcano plots representing, for each gene detected, the log_2_ fold change (x-axis) difference of animals with small relative to large social network sizes and the corresponding -log_10_ adjusted *p*-value (y-axis) in the prefrontal cortex (**a**), hypothalamus (**b**) and hippocampus (**c**) for females. For males, only the prefrontal cortex is shown (**d**) as males had either very few or no genes that were differentially expressed. Genes that were differentially expressed and upregulated in animals with small relative to large social network size are represented in red and those that were differentially expressed and downregulated in this comparison are represented in green. All other genes are represented in blue

In females, 59 genes were differentially expressed in the prefrontal cortex, 37 in the hypothalamus and 84 in the hippocampus (Fig. 3). In males, no differentially expressed genes were detected in the hippocampus, only 2 in the prefrontal cortex (*Gm13453* and *Xist*) and 1 in the hypothalamus (*Gm13453*).

**Fig. 4:**
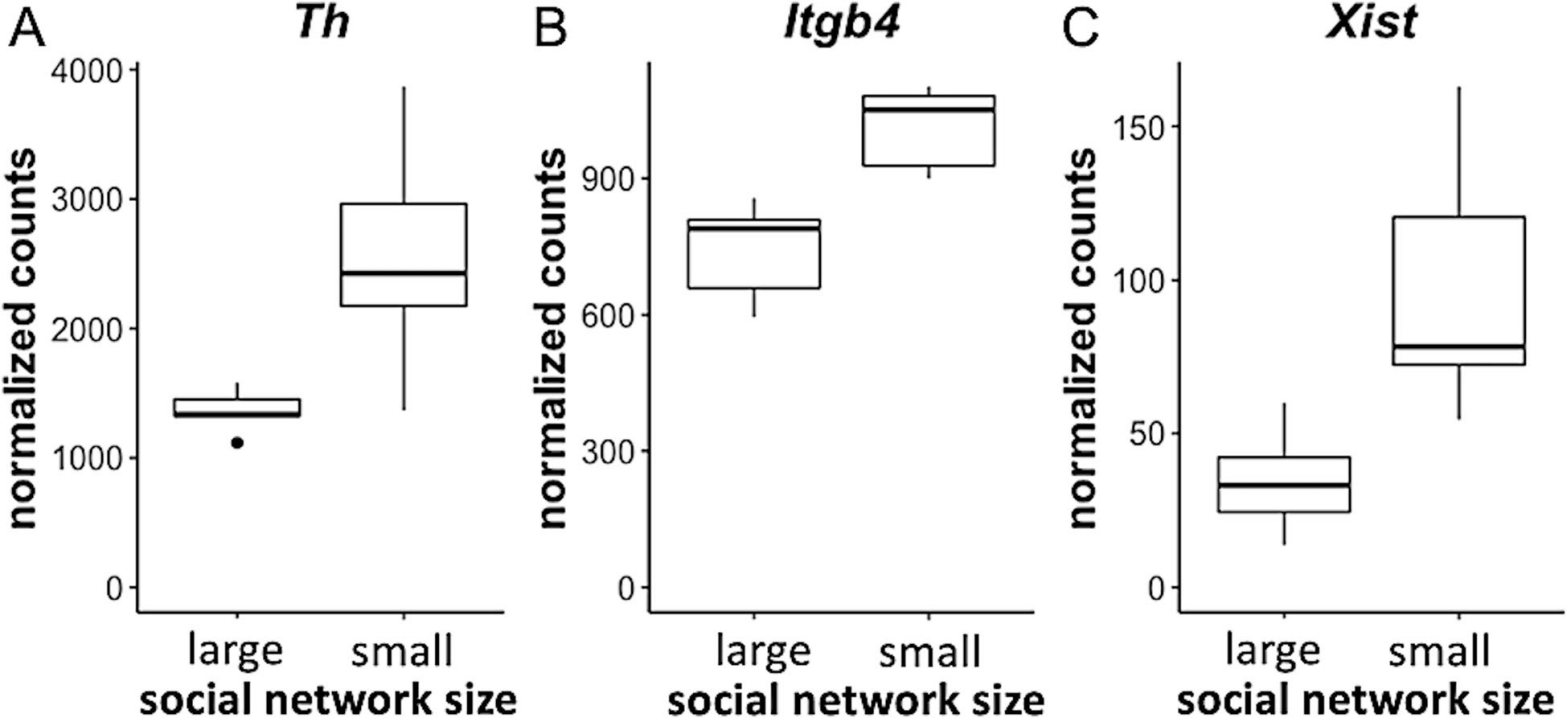
Expression levels of top differentially expressed genes highlighted during GO term analysis. Expression level is represented using DESeq2 normalized counts of top differentially expressed genes detected during enrichment analysis in the hypothalamus (**a**) and hippocampus (**b**) of females, and in the prefrontal cortex of males (c), with extreme social network sizes. The middle band within the box and whiskers plots represents the median, the bottom and top of the box represent the first and third quartiles and the whiskers denote the 95% confidence interval of the data. *Th*: Tyrosine hydroxylase; *Itgb4*: Integrin β4; *Xist*: Inactive X specific transcript

All of the results from the enrichment analysis of genes differentially expressed in animals with small relative to large social network sizes can be found in Table S3. In males, the enriched Gene Ontology (GO) terms were all related to dosage compensation by inactivation of the X chromosome in the prefrontal cortex and included only one upregulated transcript, *Xist*. In females, differentially expressed genes (DEGs) in the prefrontal cortex were not enriched for any GO terms. In the hypothalamus, DEGs upregulated in females with small network sizes were enriched most significantly for GO terms related to dopamine/catecholamine biosynthesis and metabolism (upregulated DEGs: *Th*, *Ddc*, *Cyp2d22*), followed by amine transport (upregulated DEGs: *Th*, *Ddc*, *Slc18a2*, *Chrna6*). In this same brain region, downregulated DEGs were most significantly enriched for terms related to the regulation of the inflammatory response and included genes such as *Snx4* and *Cd47*. In the hippocampus, upregulated DEGs were enriched for only one GO term for ‘proteinaceous extracellular matrix’ (DEGs: *Wnt3*, *Itgb4*, *Dmp1*, *Gpc2*, *Prelp* and *Emilin3*). The most significant enrichment terms associated with downregulated genes in the hippocampus involved mostly ion channel activity (DEGs: *Itgav*, *Slc26a8*, *Cacna2d1* and *Gabrg3*). The expression level of the top differentially expressed genes within the most significant pathways in each brain area is represented in Fig. 4.

**Fig. 5:**
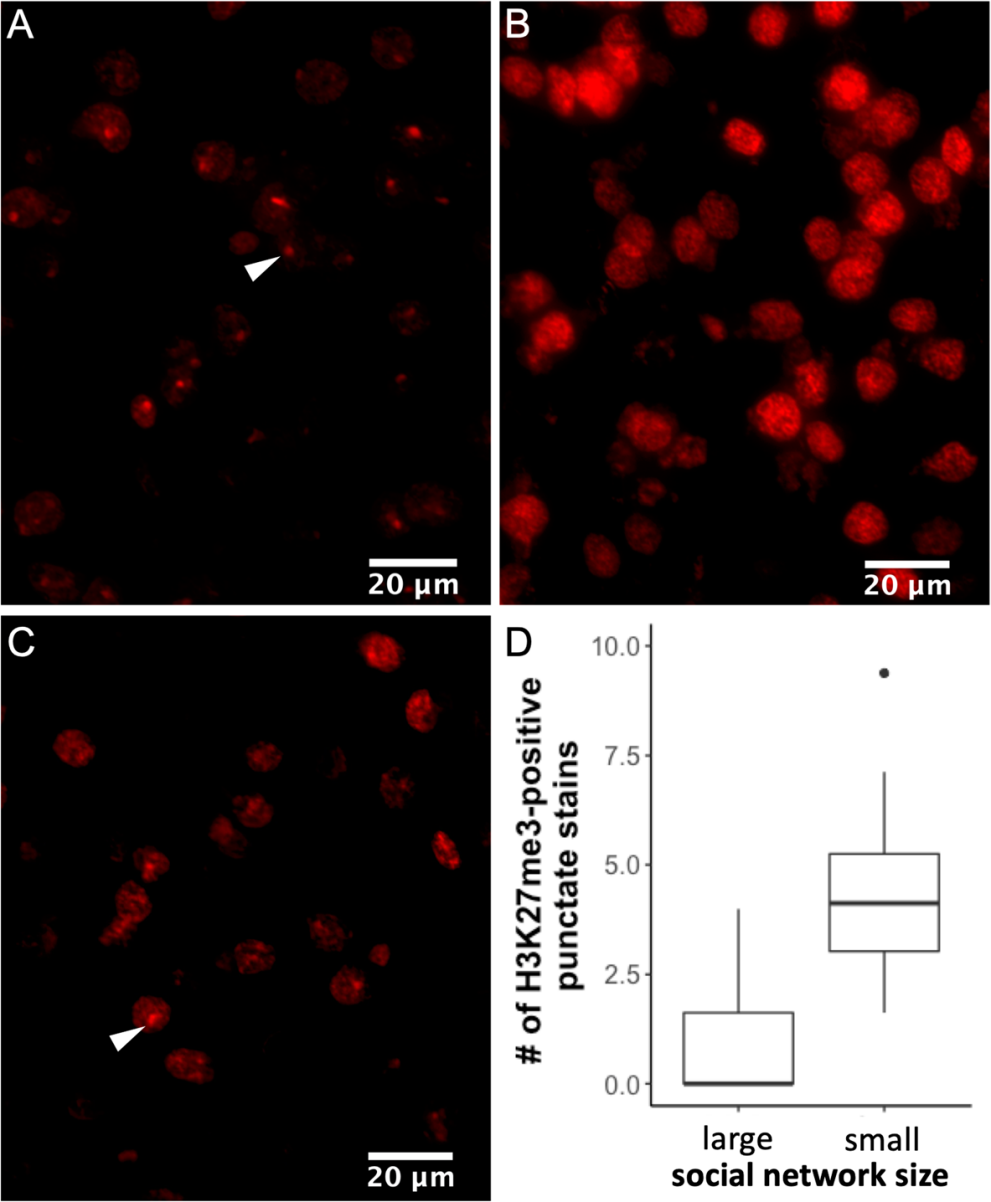
**a** Representative photograph of female prefrontal cortex showing positive punctate staining for H3K27me3 (arrowhead). Representative photographs of prefrontal cortex of males with large (**b**) and small (**c**) social network sizes stained for H3K27me3. In B, it is possible to see that the staining is diffuse and not punctate. In C, the arrowhead indicates one example of punctate staining. **d** Boxplots of average counts of H3K27me3-positive punctate stains in the prefrontal cortex of males with large and small social network sizes

Expression levels of *Xist* were low in males (mean = 66.8 counts; as a reference, this is about 479 times lower than in females, where mean = 31,975 counts). As such, to understand whether the differences in *Xist* expression in males of different social network sizes reflected differences in X-chromosome inactivation patterns, we stained brain slices of the prefrontal cortex for an epigenetic marker of the inactive X-chromosome. This marker, the Histone H3 trimethyl-lysine 27 (H3K27me3) modification, has been shown to co-localize with Xist RNA in mice [39]. We found that the number of H3K27me3-positive punctate stains differed significantly between males with large and small social network sizes (Welch’s t-test, *t* = − 3.2842, *p* = 0.0067, df = 11.735; Fig. 5). As a reference, on average, females had 15x more punctate stains than males in the same region (mean for females = 45.4 ± 3.58; mean for males = 2.97 ± 0.72).

## Discussion

In this study, we collected brains from wild house mice exhibiting extreme patterns of social interactions (i.e., on the tails of a distribution of social network sizes for the population) and tested whether these individuals showed differences in gene expression patterns in brain regions important for social behaviors. We found that, while females of contrasting social network sizes differed in the expression of hundreds of genes, males exhibited very few gene expression differences. This sex difference in number of differentially expressed genes may be due to the fact that, in males, large social networks sizes were not as extreme as in females. While sampled females with large network sizes had on average 38.1 interaction partners per day (which is 12 partners above the population mean of 26 ± 8.6 partners/day), we did not find males that were above 1 STD of the mean social network size value for the population (mean partners/day for sampled males with large social network sizes was 32.5). The large social network sizes observed in males may therefore not be sufficiently extreme to allow for the detection of gene expression differences relative to males of low social network sizes. Alternatively, or in addition to this reason, the few genes differentially expressed between males with different social network sizes could have strong effects, which is discussed later.

In the hypothalamus of females with small social network sizes, the most important pathway that contained upregulated genes relative to females with large social network sizes was related to dopamine biosynthesis, which suggests that females with fewer social partners produce more dopamine in this brain region. Dopaminergic signaling plays important roles in modulating a variety of functions/behaviors across vertebrates, such as motivation, reward, associative learning, same-sex and opposite-sex partner preference, and sexual behaviors [15, 40]. Even though dopamine levels can also be associated with maternal responses to pups or pup cues [41–43], we can exclude this possibility here as no pups were around during the time when we sampled animals for this study. Dopamine activity in certain hypothalamic nuclei has been associated with increases in aggressive responses in male rodents (e.g., [44–46]; reviewed in [47–49]). While the neurobiology of aggression has been less studied in female rodents, hypothalamic nuclei are also involved in female aggression [47–49]. Increased aggression could be one reason for which certain females in our study live in smaller social group sizes. Another possibility would be differences in social status. Naked mole-rat queens (dominant reproductive females) have significantly higher tyrosine hydroxylase (*Th*) and vesicular monoamine transporter (*Slc18a2*) gene expression in the hypothalamus than subordinate non-breeding animals of either sex [50]. These results parallel our findings in the hypothalamus of females, which may be an indication that females with small social network sizes are dominant to females with large ones, even outside of the reproductive season. Some of these effects could be exerted through the pituitary hormone prolactin, as a critical function of dopamine released from the hypothalamus is in the suppression of pituitary secretion of prolactin [51] and because, among its many functions [52], prolactin is associated with social behaviors in several taxa, including parental behaviors [53, 54] and prosocial and affiliative behaviors [55, 56]. In terms of pathways containing downregulated genes, the ones highlighted in our GO term enrichment analysis were mostly related to the regulation of the inflammatory response, and the genes repeatedly represented in those pathways were *Snx4* and *Cd47*. SNX4 is involved in endocytosis and other aspects of intracellular trafficking [57, 58] and CD47 is involved in a variety of functions, including leukocyte signaling pathways, migration and phagocytosis (reviewed in [59]), as well as axon extension [60]. It is unclear how SNX4 could relate to social network size, but the same pattern of expression differences for this gene were also observed in the prefrontal cortex of females. One link is that dysfunction in sorting nexins (the family of proteins to which SNX4 belongs) has been associated with neurogenerative diseases [61]. For instance, brain tissue from patients with (and mouse models of) Alzheimer’s disease, a disease characterized by symptoms that can affect social interactions, such as impaired cognition and memory loss, showed altered expression of SNX4 [62]. On the other hand, more direct links exist between CD47 and behavior. One study found that CD47 knockout mice exhibit significant lower sociability than wild-type littermates [63]. A separate study uncovered a main effect of acute restraint stress in puberty in reducing expression of *Cd47* in the hippocampus and the prefrontal cortex [64]. Combined, the current results and those from previous studies seem to highlight CD47 as a molecule deserving more studies in the context of social behavior.

The hippocampus was the region with the largest number of differentially expressed genes between females with opposing social network sizes. A number of genes related to the proteinaceous extracellular matrix (ECM) were upregulated in the females with small social network size. The ECM is a structure that surrounds the cells. In the central nervous system, the ECM affects chemical communication between neurons and it has been proposed that the ECM has an important role in regulating both synaptic and homeostatic forms of plasticity not only during development, but also in adulthood (reviewed in [65]). Experimental alterations of the hippocampal ECM, for instance through enzymatic removal, have been shown to impact memory and learning [66], which are two faculties that could affect the ability to establish or maintain social relationships. In addition to these roles, some of the ECM genes highlighted in the enrichment analysis, such as *Wnt3*, *Gpc2* and *Itgb4*, are also involved in adult hippocampal neurogenesis [67, 68] and in abnormal behavior (e.g., hyperlocomotion in ITGB4 knockout). Winning fights has been associated with increased hippocampal neurogenesis in mice [69], which is consistent with the idea proposed earlier that females with small social network sizes in our study may be more aggressive and potentially better at fighting than counterparts with large social network sizes. It may be possible that females that are better at fighting are better capable of protecting their territories, therefore maintaining smaller social groups. It has been previously demonstrated in rhesus macaques (*Macaca mulatta*) that brain structure and functional connectivity are affected by social network size [70]. Structural brain changes involving plasticity and neurogenesis may therefore be necessary for dealing with, and/or a consequence of, living in larger social groups.

While in females no major pathways were found in the prefrontal cortex, in males this was the only region where a pathway was detected that differentiated animals with small and large social network sizes. This pathway was related to the inactivation of the X chromosome. This is unusual, because inactivation of the X chromosome is a mechanism of dosage compensation mainly found in female mammals, whereby one X chromosome is inactivated thereby ensuring the same dosage in males and females (as males only have one X chromosome and females have two). X inactivation in males is usually restricted to spermatogenesis, and *Xist* transcription usually only found in the testis [71]. Expression of the long non-coding RNA Xist by the X chromosome that will become inactive is thought to initiate the X inactivation process (reviewed in [72–74]). Recently, however, it has been suggested that *Xist* expression itself is insufficient to inactivate the X chromosome in males but that it does silence X-linked genes in females [75]. The presence of Xist RNA coating the future inactive chromosome recruits complexes responsible for trimethylation of lysine 27 on histone H3 (H3K27me3) [76] and this epigenetic marker can be visualized using specific antibodies, producing a punctate staining in cell nuclei when present [39]. When we used this approach to understand whether the changes in *Xist* expression suggested X inactivation, we did observe more cells showing punctate staining in males with small relative to large social network sizes but this in no way approached female levels, which were 15x higher.

How could *Xist* expression mediate differences in social interaction behavior? One way is through its inactivation of the X chromosome. In humans, X-linked genes are involved in neurobehavioral disorders (such as fragile-X and autism spectrum disorder) [77, 78] and mice with abnormal number of X chromosomes (such as X0 or XXY) also show altered social behaviors relative to their counterparts with regular numbers of X chromosomes, regardless of gonadal sex [79]. While we do find evidence of some level of X inactivation in the males with small social network size, we think this is unlikely to be the major cause of behavioral changes, as we did not find other X-linked genes (besides *Xist*) to be greatly different between males with different social network sizes. Several lines of evidence point towards *Xist* having other functional roles. *Xist* is overexpressed in cells of female patients with either bipolar disorder or major depressive disorder relative to healthy females [80]. Long noncoding RNAs, such as Xist, have been found to interact with other types of RNA in ways that, when dysregulated, may contribute to neurodegenerative disorders and cancers [81, 82]. In particular, *Xist* seems to interact with microRNAs to affect the progression and development of lung, pancreatic and prostate cancers [83–85] and also to potentially impact neurogenerative disorders [86, 87]. For instance, a study using in vitro and in vivo mouse models of Alzheimer’s disease showed an increase of Xist RNA expression in the hippocampus relative to controls [86]. Silencing of *Xist* in that study led to an upregulation of a microRNA signaling pathway (miR-124/BACE1). This ability to interact with microRNAs could functionally result in a modified abundance of microRNAs and, consequently, alter expression of other genes post-transcriptionally. In other words, changes to *Xist* expression could have major effects on the formation of certain peptides, without alteration to the expression levels of the genes coding for those peptides.

## Conclusions

In conclusion, we detected differences in neural gene expression between mice with extreme differences in social network sizes. While our set up does not allow us to differentiate between whether social interactions led to the differences in gene expression profiles or are a consequence of these, our data reveals several candidate genes that may be associated with social network size. The array of genes detected differed by sex, which suggests that there may be different reasons underlying differences in social network size between the sexes, even during the non-reproductive season. Our study shows that, even in “noisy” conditions that underly studying wild animals in their natural environment, we are still able to detect genes associated with social behavior. We highlight genes that are potentially understudied in the context of social behaviors and hope that further research will elucidate the precise mechanisms by which these genes may be linked to social behaviors.

## Methods

### Wild house mouse population

The population of wild house mouse (*Mus musculus domesticus*) used for this research is part of a long-term study initiated by Barbara König and colleagues in 2003 (the history and detailed description can be found in [88]). The study site consists of a 72 m^2^ barn located in the vicinity of Zurich, Switzerland. The barn is an open space, structured into 4 sectors by four 75 cm high dividers. The large dividers and the walls of the barn contain holes, allowing the mice to access not only the entire space inside the building, but also to freely move in and out of the building. The wall holes are not large enough for large predators, such as cats, foxes and owls that regularly occur around the barn. Water and food are provided ad libitum in water bottles and food trays distributed in equal numbers throughout the sectors. Nest building materials, such as straw and hay, are also made available weekly and dispensed throughout the barn.

### Automated social interaction tracking system

The automated RFID-based recording system used in this study has been described in detail in [37]. In brief, the system consists of 40 artificial nest boxes (15 cm in diameter each; 10 per sector of the barn) with two RFID-reading antennas fitted around a cylindrical tube allowing access to the box. The antennas read RFID tags (Trovan ID-100, Euro ID Identifikationssysteme GmbH & Co, Germany), that are implanted subcutaneously into every individual captured in the population upon reaching 18 g. Therefore, as tagged mice go in or out of the nest box, their identities, along with a timestamp, are automatically logged into a computer located inside the barn. Each day, the information on all mouse movements through the antennas is transferred to a central database. Using this data, we can determine, per logged individual, how many other individuals they overlapped in time with when inside any given nest box [89].

For the current study, we analyzed brain gene expression from house mice whose tissues had been previously used in a study aimed at identifying relationships between immune system functioning and social behavior [90]. Those animals were captured during the non-reproductive season (winter months), which reduces the presence of untagged individuals (pups or subadults below 18 g), as well as any confounding effects due to reproductive activity. Also, during this season, mice in our population spend a considerable amount of time (up to 19 h per day) inside of the nest boxes [89]; therefore, we consider the number of partners encountered in nest boxes during this time as a good proxy of their social network size. Animals with extreme social network sizes were selected based on being consistently (over 3 separate non-consecutive days during the week prior to capture) approximately either 1 standard deviation (STD) above (large social network size) or below (small social network size) the mean egocentric social network size for the entire population (mean ± STD: 26 ± 8.6 partners/day) [90]. Social network size is defined here as the total number of different individuals encountered in nest boxes per day; in social network literature, this value (number of ties) is referred to as degree or egocentric network size [91]. Social network size values presented in the text for the focal animals represent the average social network size over the 3-day period mentioned above. Sample sizes were 7 for females of each type (large or small) of social network size, and 7 and 8 for males with large and small social network sizes, respectively. Average social network size was not different between the sexes for the sampled animals [90]. Estimated age (in days) of sampled animals did not significantly differ by sex or social network size type (large or small) and was 207.2 ± 57.7 and 246.6 ± 68.1 for females and males with large social networks, respectively, and 250 ± 63.2 and 125.8 ± 45.6, for females and males with small social networks, respectively [90]. Body mass was obtained using a Sartorius scale (BL1500S) and differences in body mass due to sex, social network size or an interaction of the two terms, were tested using a two-way anova in R. As the RFID-logging system also allowed us to track how much time animals spent in nest boxes and how often they went in and out of boxes, we used these metrics to assess whether time spent in nest boxes or activity (going in and out of nest boxes) explained variation in the social network size trait. To do this, we used a linear regression to test whether social network size varied as a function of either time spent in boxes or activity, using data from a 24 h period for the entire population. Finally, we estimated repeatability of the social network size trait for mice in our population during the non-breeding season using social network size values obtained in the same way as detailed above, for 4 separate dates spaced out by approximately 1 week, collected at a different time from when this experiment was taking place. Repeatability (R), confidence interval and the significance of repeatability were estimated using the rptR package [92] in R, using the Poisson function. The confidence intervals were obtained by performing 1000 parametric bootstrap runs and the *p*-value was obtained by using 1000 permutations. While not explicitly controlling for the nonindependent nature of social network data, this method allows for easy comparison with other studies as it is routinely used to estimate repeatability of different traits, including behavioral traits (see [93] for a meta-analysis).

### Brain collection and dissection of brain regions

Target animals, selected based on their social network size values as described in the previous section, were captured within 2 h of sunrise by blocking the entrance to the nest boxes they used with most frequency. The nest boxes were inspected sequentially by opening the entrance to the box and allowing animals to exit into a glass jar. When a target animal was identified in the glass jar using a handled RFID reader, it was brought to a processing station at the barn, weighed and euthanized via CO_2_ inhalation. The brain was extracted from the skull and immediately placed on dry ice over a piece of foil. The brains were later transferred to a − 80 °C freezer until further processing.

Brains were coronally sectioned at − 18 °C on a Leica CM1860UV cryostat. Surgical micropunches (EMS Rapid Core Instruments) were used to dissect the brain regions of interest from 100 μm slices, which were spaced out by two 20 μm slices that were collected for histochemistry onto microscope slides (Fisherbrand, item 12–550-15). We collected punches for the prefrontal cortex, the hypothalamus and the entire hippocampus following the coordinates described in [90] based on the Allen Mouse Brain Atlas: P56, Coronal Reference Atlas [94]. We focused on these brain regions because they include nuclei important for social decision making and social memory [14, 95]. Punches from each of these brain regions were preserved in separate tubes for each region, each containing 1 mL of Trizol reagent (Ambion, item 149,204) and zirconium 1.5 mm size beads (Benchmark Scientific, item D1032–15). Tissue homogenization was performed by agitating the tubes for 20 s at a 4 m s^− 1^ speed (Beadbug 6 homogeneizer, Benchmark Scientific), followed by a 5 min rest period. The liquid was transferred into a new tube and preserved at − 80 °C until the RNA isolation procedure.

### RNA isolation, library preparation and sequencing

Total RNA was isolated from the aqueous layer obtained post chloroform extraction, using the Direct-zol RNA Miniprep Plus kit (Zymo, catalogue # R2071) according to manufacturer’s instructions, with the additional DNase I in-column treatment step. RNA samples were sent to Novogene Corporation Inc. (Chula Vista, CA, USA) where RNA quantity, integrity and purity were assessed on an Agilent 2100 Bioanalizer (Agilent Technologies, Santa Clara, CA) and cDNA libraries (250 ~ 300 bp inserts; NEBNext® UltraTM RNA Library Prep Kit; New England BioLabs, Inc., Ipswich, MA, USA) and paired-end sequencing of libraries (PE150; Illumina Novaseq 6000) were performed according to standard protocols. Only one sample had a RIN value below 7 (at 6.2; hypothalamus of Mus19, a female with large social network size) but none of the post-sequencing quality controls or differential gene expression results suggested that this sample acted as an outlier and so this sample was not removed from the analysis. RNA extraction failed from one sample (hypothalamus of Mus18, a male with small social network size). Sample sizes for hypothalamic sequencing data were therefore different than that for other brain regions and included 7 for animals with large and small social network sizes for each sex. Establishing the appropriate sample size to use in any given RNA-seq experiment is far from straightforward. We used the guidelines and results provided by [96] to determine the appropriate sample size for our experiment with the aim of detecting ≥ 80% of differentially expressed genes at a wide range of fold change differences. An average of 120 million paired-end raw reads were obtained for each sample.

### Mapping and differential gene expression analysis

On average, 94.5% of clean (post adapter removal and quality filtering) reads were mapped to the mouse reference genome [*Mus musculus* (GRCm38/mm10)] using STAR [97], representing an average of 116 million mapped reads per sample (full information on mapping statistics per sample can be found in Table S1). To count the number of mapped reads to each gene, HTSeq was used. Differential expression analysis of pairwise comparisons of the animals with small relative to animals with large social network size within each sex was performed using the DESeq2 R package [98, 99]. To control for the false discovery rate due to multiple testing, *p*-values were adjusted using the Benjamini-Hochberg procedure. Genes were considered as statistically differentially expressed when adjusted *p*-values were < 0.05. The function plotPCA within the DESeq2 package was used on variance stabilized transformed (VST) count data to prepare the PCA graph. Heatmap visualization of differentially expressed genes was prepared in Heatmapper [100], using DESeq2 normalized read counts and complete linkage as clustering method and Pearson as distance measurement method. Boxplots for a subset of the differentially expressed genes were prepared using the ggplot2 R package [101].

### GO enrichment analysis of differentially expressed genes

Enrichment analysis of differentially expressed genes was performed in clusterProfiler v2.4.3. *P*-values were adjusted as above, using the Benjamini-Hochberg procedure. GO terms with adjusted *p*-values of < 0.05 were considered significant.

### Immunofluorescence and microscopy

To understand whether the gene expression differences found for *Xist* in the prefrontal cortex of male mice with small relative to large social network size actually reflected symptoms of X-chromosome inactivation differences, we used immunofluorescence on the adjacent slices that were obtained during brain dissection, which had been stored at − 80 °C. We used an epigenetic marker of the inactive X-chromosome, the Histone H3 trimethyl-lysine 27 (H3K27me3) modification, which co-localizes with Xist RNA [39]. The antibody used was rabbit anti-tri-methyl-histone H3 K27-3 m (1:1000, Cell Signaling catalogue #9733S), which has been previously validated and used in mice under similar immunofluorescence conditions [39, 102]. The secondary fluorescently labeled antibody was Alexa Fluor 568-conjugated goat anti-rabbit (1:1000; Life Technologies catalogue #A11011).

Slides were brought to room temperature and tissue was outlined with a hydrophobic barrier (ImmEdge Hydrophobic Barrier PAP Pen, Vector Labs catalogue #H-4000) before being exposed to cold 4% paraformaldehyde (prepared in 1x PBS) for 20 min. Slides were then rinsed 3 times in 1x PBS and blocked in 1x PBS containing 2% normal goat serum and 0.2% Triton-X for 1 h. Incubation in primary antibody took place over two nights at 4 °C in a humid chamber. Slides were then rinsed 3 times in 1x PBS containing 0.2% Triton-X and exposed to the secondary antibody for 90 min. After rinsing 3 times in 1x PBS, the slides were allowed to dry and coverslipped with DAPI Fluoromount-G (SouthernBiotech, Birmingham, AL).

For each animal, four brain slices containing the prefrontal cortex region were photographed on a Zeiss Imager.M2, with an Axiocam 506 mono camera. Per slice, two photographs were taken with a 40x objective, one for each hemisphere. The number of H3K27me3-positive punctate stains (marking the inactive X-chromosome) in the entire field of each photograph was counted by hand by an observer blind to the treatments. The number of DAPI-positive stained nuclei for the same field was counted automatically, using the ZEN 2.3 (blue edition) software. Numbers of H3K27me3-positive punctate stains and DAPI-positive nuclei were averaged per animal. We first tested whether the average number of DAPI stained nuclei in the prefrontal cortex differed by sex or social network size trait using a two-way ANOVA. As no effects of sex, social network size trait or their interaction were found (Table S4), we used the average counts of H3K27me3-positive punctate stains for the subsequent analysis, rather than a ratio of H3K27me3-positive punctate stains per DAPI-positive nuclei. We compared H3K27me3-positive punctate stains in males with large social network size to those with low social network size using a Welch’s t-test. Boxplots for these results were prepared using the ggplot2 R package [101].

## Supplementary information

**Additional file 1 Table S1.** RNA-Seq mapping statistics. **Table S2.** Differentially expressed genes between animals with large and small social network size in females and males in the different brain regions. **Table S3.** Statistically significant GO (Gene Ontology) terms . Abbeviations for ontology categories: BP - Biological Processes; CC - Cellular Component; MF - Molecular Function. **Table S4.** Two-way ANOVA table for test of differences in number of DAPI-positive nuclei in the prefrontal cortex of mice. **Figure S1.** Social network size as a function of (A) total length of stay in nest boxes and (B) number of times entering/exiting nest boxes. **Figure S2.** Body mass of sampled animals.

## Data Availability

The datasets generated and/or analyzed during the current study are available in the NCBI Gene Expression Omnibus (GEO) repository, with record GSE148075 (https://www.ncbi.nlm.nih.gov/geo/query/acc.cgi?acc=GSE148075). The reference mouse genome used was the GRCm38/mm10 assembly and it was obtained from the University of California Santa Cruz (UCSC) Genomics Institute website (https://genome.ucsc.edu/). The accession numbers/gene IDs in Table S2 are from Ensembl (https://www.ensembl.org/Mus_musculus/Info/Index). The Gene Ontology (GO) IDs in Table S3 are from the Gene Ontology database (http://geneontology.org/).

## Abbreviations

ANOVA: Analysis of Variance
BACE1: Beta-secretase 1 or beta-site amyloid precursor protein cleaving enzyme 1
Chrna6: Cholinergic receptor, nicotinic, alpha 6
Cd47: Cluster of differentiation 47
Cyp2d22: Cytochrome P450, family 2, subfamily d, polypeptide 22
cDNA: Complementary deoxyribonucleic acid
Cacna2d1: Voltage-dependent calcium channel subunit alpha-2/delta-1
DAPI: 4′,6-diamidino-2-phenylindole
DEG: Differentially expressed genes
DNMT1: DNA (cytosine-5)-methyltransferase 1
Ddc: Aromatic-L-amino-acid decarboxylase
Dmp1: Dentin matrix acidic phosphoprotein 1
ECM: Extracellular matrix
Emilin3: Elastin microfibril interface-located protein 3
Gabrg3: Gamma-aminobutyric acid receptor subunit gamma-3
Gpc2: Glypican-2
H3K27me3: Trimethylation at lysine 27 of histone H3
Itgav: Integrin alpha-V
Itgb4: Integrin beta-4
miR-124: MicroRNA 124
PBS: Phosphate based buffer
Prelp: Prolargin
RFID: Radio-frequency identification
RNA: Ribonucleic acid
RNA-seq: RNA-sequencing
Slc18a2: Synaptic vesicular amine transporter or Solute carrier family 18 member 2
Slc26a8: Testis anion transporter 1 or Solute carrier family 26 member 8
Snx4: Sorting nexin-4
STD: Standard deviation
Th: Tyrosine hydroxylase
V1aR: Vasopressin V1a receptor
Wnt3: Proto-oncogene Wnt-3
Xist: X inactive specific transcript
